# Activity, Abundance, and Localization of Quorum Sensing Receptors in *Vibrio harveyi*

**DOI:** 10.3389/fmicb.2017.00634

**Published:** 2017-04-18

**Authors:** Nicola Lorenz, Jae Yen Shin, Kirsten Jung

**Affiliations:** Microbiology, Munich Center for Integrated Protein Science (CIPSM) at the Department of Biology I, Ludwig-Maximilians-Universität MünchenMartinsried, Germany

**Keywords:** phosphorylation, fluorescence microscopy, marine bacteria, qRT-PCR, autoinducer, hybrid histidine kinase, response regulator, histidine phosphotransfer protein

## Abstract

Quorum sensing (QS) is a process enabling a bacterial population to communicate via small molecules called autoinducers (AIs). This intercellular communication process allows single cells to synchronize their behavior within a population. The marine bacterium *Vibrio harveyi* ATCC BAA-1116 channels the information of three AI signals into one QS cascade. Three receptors perceive these AIs, the hybrid histidine kinases LuxN, Lux(P)Q and CqsS, to transduce the information to the histidine phosphotransfer (HPt) protein LuxU via phosphorelay, and finally to the response regulator LuxO. Hence, the level of phosphorylated LuxO depends on the AI concentrations. The phosphorylated LuxO (P-LuxO) controls the expression of small regulatory RNAs (sRNAs), which together with the RNA chaperon Hfq, destabilize the transcript of the master regulator *luxR*. LuxR is responsible for the induction and repression of several genes (e.g., for bioluminescence, exoprotease and siderophore production). *In vivo* studies with various mutants have demonstrated that the ratio between kinase and phosphatase activities of the individual QS receptors and therefore the P-LuxO/LuxO ratio is crucial not only for the output strength but also for the degree of noise. This study was undertaken to better understand the inherent design principles of this complex signaling cascade, which allows sensing and integration of different signals, but also the differentiated output in individual cells. Therefore, we quantitatively analyzed not only the enzymatic activities, but also the abundance and localization of the three QS receptors. We found that LuxN presents the highest capacity to phosphorylate LuxU, while the phosphatase activity was comparable to LuxQ and CqsS *in vitro*. In whole cells the copy number of LuxN was higher than that of LuxQ and CqsS, and further increased in the late exponential growth phase. Microscopy experiments indicate that LuxN and LuxQ form independent clusters. Altogether, these results suggest, that the three QS receptors act in parallel, and *V. harveyi* has developed with LuxN the most dynamic sensing range for HAI-1, the species-specific AI.

## Introduction

**Figure 1:**
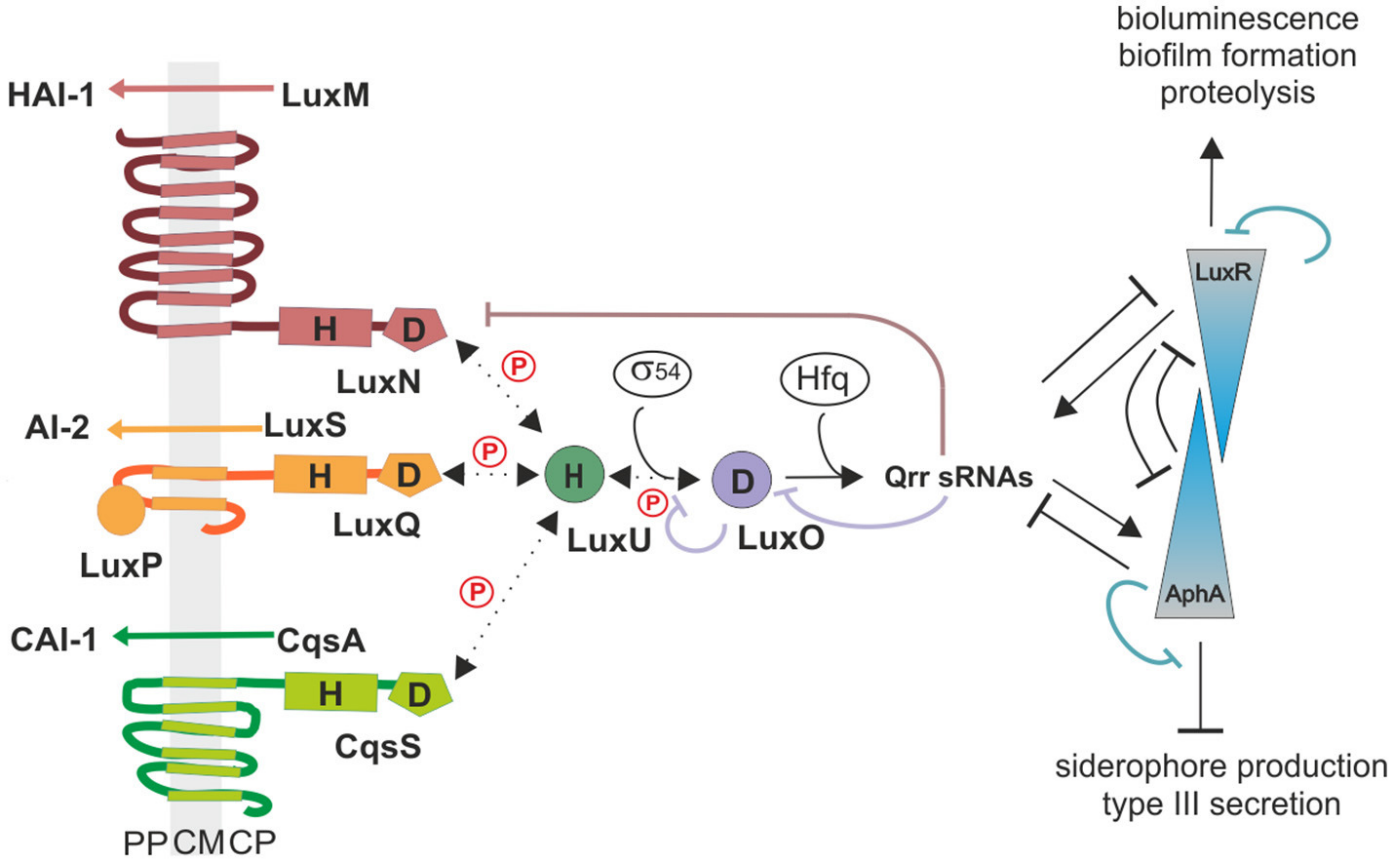
**The QS cascade of *V. harveyi*.** LuxM, LuxS, and CqsA synthesize three different AIs (HAI-1, AI-2, and CAI-1). At low cell density (LCD) and low AIs concentrations, the receptors autophosphorylate and transfer the phosphoryl group via phosphorelay to the HPt protein LuxU, and subsequently to the response regulator LuxO. The phosphorylated LuxO (P-LuxO) together with σ^54^ induces the expression of five regulatory small RNAs (Qrr 1-5). These sRNAs along with the chaperon Hfq, destabilize the transcript of the QS master regulator LuxR. At high cell density (HCD) and high AIs concentrations, binding of the AIs to the corresponding hybrid histidine kinases LuxN, LuxQ (together with the periplasmic binding protein LuxP), and CqsS inhibit the kinase activities. Thereby the phosphate is drained from the cascade and LuxR is produced. LuxR induces the expression of genes required for bioluminescence, biofilm formation and proteolysis, and represses genes for siderophore production and type III secretion systems. AphA is present at LCD and the counterpart of LuxR. H indicates histidine and D aspartate which are phosphorylation targets. PP, periplasm; CM, cytoplasmic membrane; CP, cytoplasm; Dotted arrows indicate phosphotransfer and continuous arrows show transcriptional or posttranslational regulatory interactions.

*Vibrio harveyi* ATCC-BAA 1116 (reclassified as *V. campbellii*; Lin et al., 2010) is a marine, free-living bacterium, which can be found on the surface of algae or as pathogen in shrimp or fish. One outstanding characteristic of this Gram-negative microorganism is its ability to produce bioluminescence in a cell density dependent manner called quorum sensing (QS). This communication process allows cells to coordinate their behavior within a population by altering gene expression upon a threshold concentration of signaling molecules (Keller and Surette, 2006). *Vibrio harveyi* responds to three different classes of autoinducers (AIs) and the information is channeled into one phosphorelay cascade. The first AI is HAI-1 an acyl-homoserine lactone [*N*-(3-hydroxybutyryl)-homoserine lactone], which is produced by the synthase LuxM and is a signaling molecule specific for *V. harveyi* (Cao and Meighen, 1989). The second one is AI-2, a furanosyl borate diester, synthesized by LuxS and can be considered as a global signaling molecule since it is produced by various bacterial species (Chen et al., 2002). The third one is CAI-1, a long-chain amino ketone [*(Z)* 3-aminoundec-2-en-4-one] (Ea-C8-CAI-1), produced by CqsA and is specific to members of the *Vibrio* genus (Ng et al., 2011). Interestingly, these AIs follow a distinct synthesis pattern, the concentration of each AI differs in accordance with the growth phase (Anetzberger et al., 2012). While the concentration of AI-2 increases during the exponential growth phase, HAI-1 and CAI-1 can be detected only at the late exponential phase (Anetzberger et al., 2012). Each of the AIs, HAI-1, AI-2, and CAI-1, are perceived by three different receptors, the hybrid histidine kinases LuxN, LuxQ (together with the periplasmic binding protein LuxP) and CqsS, respectively (Figure 1). These membrane-bound receptors comprise a transmitter domain, containing a dimerization and histidine phosphotransfer domain (DHp) and a catalytic and ATP-binding (CA) domain including the conserved histidine residue. Hybrid histidine kinases also contain a C-terminal receiver domain harboring a conserved aspartate residue.

At low cell density (LCD) and low AIs concentration, the receptors act as kinases. Specifically, the conserved histidine residue of the receptors gets autophosphorylated to subsequently transfer the phosphoryl group to their conserved aspartate. Next, this phosphoryl group gets transferred to the histidine phosphotransfer (HPt) protein LuxU, which in turn phosphorylates the response regulator LuxO at its conserved aspartate residue (Freeman and Bassler, 1999). P-LuxO is activated and together with the sigma factor σ^54^ induces the transcription of five small regulatory RNAs (Qrr1-5) (Lenz et al., 2004). These sRNAs and the RNA chaperone Hfq act together to ultimately destabilize and degrade the mRNA of the master regulator *luxR*, which regulate the QS phenotypes by maintaining it in the OFF state (Tu and Bassler, 2007). At high cell density (HCD), AIs reach higher concentrations and bind to the corresponding receptors, which inhibits their kinase activities. Consequently, LuxU gets dephosphorylated and thus, the phosphoryl groups are drained from the cascade (Timmen et al., 2006). Because of this LuxO inactive form, the downstream cascade to induce Qrr1-5 is inhibited. LuxR is produced and induces the expression of genes responsible for bioluminescence (Bassler et al., 1994), biofilm formation (Anetzberger et al., 2009) and represses genes encoding a type III secretion system (Henke and Bassler, 2004) as well as siderophores (Lilley and Bassler, 2000). Moreover, the QS cascade in *V. harveyi* also comprises five feedback loops: LuxO and LuxR regulate negatively their own transcription by binding to the corresponding promoter regions (Chatterjee et al., 1996; Tu et al., 2010). LuxR directly activates the transcription of the sRNAs (Tu et al., 2008). The sRNAs in turn control *luxO* mRNA via sequestration (Feng et al., 2015). Furthermore, the translation of *luxMN* is negatively controlled by the sRNAs (Qrr 1-5) (Teng et al., 2011). Finally, the transcription factor AphA, another master regulator, is induced at LCD and induces the expression of Qrr sRNAs (Rutherford et al., 2011; Feng et al., 2015).

It was shown recently that the ratio between the kinase and phosphatase activity of the hybrid histidine kinases and therefore the amount of phosphorylated LuxU/LuxO are important for the output strength and for the degree of noise (Plener et al., 2015). The pools of P-LuxU and P-LuxO determine the amount of sRNAs per cell and accordingly the copy number of the master regulator LuxR (Plener et al., 2015). Using various *V. harveyi* mutants, the impact of each subsystem was studied for QS activation at the population and single-cell level. It was found that in the presence of all three AIs, the output was homogeneous while in the absence of one or two AIs the QS activation varied from cell to cell (Plener et al., 2015). Here, we characterize the *in vitro* enzymatic activities of the QS receptors and their abundance and localization in whole cells to better understand sensing and integration of different signals, but also the differentiated output in individual cells. We found differences in their kinase but not in their phosphatase activities *in vitro*. Moreover, the copy numbers of these three receptors differ at the transcript and at the protein level. Finally, our microscopy data show that LuxN and LuxQ form clusters *in vivo*.

## Materials and methods

### Bacterial strains and growth conditions

Strains and plasmids used in this study are listed in Table 1. The *Escherichia coli* strains were aerobically grown in LB medium (10 g/l NaCl, 10 g/l tryptone, 5 g/l yeast extract) at 37°C in a rotary shaker. The *V. harveyi* strains were cultivated in autoinducer bioassay (AB) medium (Greenberg et al., 1979) or Luria marine (LM) medium (20 g/l NaCl, 10 g/l tryptone, 5 g/l yeast extract) and were grown aerobically in a rotary shaker at 30°C. When required, media were solidified by using 1.5% (w/v) agar. If necessary, media were supplemented with 50 μg/ml kanamycin sulfate and/or 100 μg/ml ampicillin sodium salt. The conjugation strain *E. coli* WM3064 was grown in the presence of 300 μM meso-diaminopimelic acid (DAP). For microscopy and in-gel fluorescence experiments *V. harveyi* cells were grown overnight in LM medium and afterwards inoculated 1:5,000 in fresh AB medium. For the in-gel fluorescence approach cells were harvested in the early or late exponential phase (OD_600_ ~0.08 and ~0.7, respectively).

**Table 1:** **Bacterial strains and plasmids used in this study**.

**Strain or plasmid**	**Relevant genotype or description**	**Reference or source**
**BACTERIAL STRAINS**		
*E. coli* DH5α-λpir	F^−^φ80d*lacZ* ΔM15 Δ (*lacZYA*-*argF*)U169 *recA1 hsdR17 deoR thi-*1 *supE44 gyrA96 relA1/*λ*pir*	Miller and Mekalanos, 1988
*E. coli* WM3064	*thrB*1004 *pro thi rpsL hsdS lacZ* ΔM15 RP4-1360 Δ(*araBAD*)567 Δ*dapA*1341::[*erm pir*(wt)]	W. Metcalf, University of Illinois, Urbana-Champaign
*E. coli* JM109	*recA1 endA1 gyrA96 traD36 thi hsdR17 supE44 λ^−^ relA1 Δ(lac-proAB)/*F' *proA^+^B^+^ lacI^*q*^ lacZΔ*M15	Yanisch-Perron et al., 1985
*E. coli* Rosetta (DE3) pLysS	F^−^*ompT hsdS*_*B*_(r_B_^−^ m_B_^−^) *gal dcm* (DE3) pLysSRARE (Cam^R^)	Novagen
*E. coli* MDAI-2	*luxS::*Tet^*r*^-derivative of *E. coli* W3110	DeLisa et al., 2001
*V. harveyi* ATCC BAA-1116	wild type	Bassler et al., 1997
*V. harveyi* ATCC BAA-1116 *ΔluxNΔluxQΔcqsS*	wild type *ΔluxNΔluxQΔcqsS*	Plener et al., 2015
*V. harveyi* ATCC BAA-1116 P*_*luxN*_*-*luxN*-*mNeonGreen*	Integration of P*_*luxN*_*-*luxN*-*mNeonGreen* at the native locus in *V. harveyi* ATCC BAA-1116	This study
*V. harveyi* ATCC BAA-1116 P*_*luxQ*_*-*luxQ*-*mNeonGreen*	Integration of P*_*luxQ*_*-*luxQ*-*mNeonGreen* at the native locus in *V. harveyi* ATCC BAA-1116	This study
*V. harveyi* ATCC BAA-1116 P*_*cqsS*_*-*cqsS*-*mNeonGreen*	Integration of P*_*cqsS*_*-*cqsS*-*mNeonGreen* at the native locus in *V. harveyi* ATCC BAA-1116	This study
**PLASMIDS**		
pGEX_LuxP	*luxP* in pGEX-4T1	Neiditch et al., 2005
pQE30LuxU-6His	*luxU* in pQE30	Timmen et al., 2006
pNKN	*luxN* in pPV5-10	Anetzberger et al., 2012
pNKQ	*luxQ* in pPV5-10	Anetzberger et al., 2012
pKK223-3 cqsS-F175C	*cqsS-F175C* in pKK223-2	This study
pNPTS138-R6KT-GFP	*mobRP4*^+^ *ori*-R6K *sacB gfp; Km^*r*^*	Lorenz et al., 2016
pNPTS138-R6KT-mNeonGreen	*mobRP4*^+^ *ori*-R6K *sacB mNeonGreen; Km^*r*^*	This study
pNPTS138-R6KT	*mobRP4^+^ ori-*R6K	This study
P*_*luxN*_*-*luxN*-*mNeonGreen*	P*_*luxN*_*-*luxN*-*mNeonGreen*; Km^*r*^	
pNPTS138-R6KT P*_*luxQ*_*-*luxQ*-*mNeonGreen*	*mobRP4^+^ ori-*R6K P*_*luxQ*_*-*luxQ*-*mNeonGreen*; Km^*r*^	This study
pNPTS138-R6KT P*_*cqsS*_-cqsS*-*mNeonGreen*	*mobRP4^+^ ori-*R6K P*_*cqsS*_-cqsS*-*mNeonGreen*; Km^*r*^	This study

### Generation of full-length protein fluorophore hybrids

Molecular methods were carried out according to standard protocols (Sambrook, 1989) or according to manufacturer's instructions. Kits for the isolation of plasmids and purification of PCR products were purchased from Südlabor (Gauting, Germany). Enzymes were purchased from New England Biolabs (Frankfurt, Germany) and Fermentas (St. Leon-Rot, Germany). Chemically competent cells of *E. coli* were transformed with the corresponding plasmids (Inoue et al., 1990). mNeonGreen (Shaner et al., 2013) (Allele Biotechnology, San Diego) was used to replace GFP in the vector pNPTS138-R6KT-GFP after restriction with SpeI and PspOMI. For construction of genes coding for the full-length receptors tagged with mNeonGreen (P_*luxN*_-*luxN*-*mNeonGreen*, P_*luxQ*_-*luxQ*-*mNeonGreen*, and P_*cqsS*_-*cqsS*-*mNeonGreen*) the coding sequence as well as promoter sequence of *luxN, luxQ*, and *cqsS* were ligated into pNPTS138-R6KT-mNeonGreen after restriction with EcoRI/BamHI (in case of *luxN*) or BamHI/PspOMI (for *luxQ* and *cqsS*) (Table S1). Chromosomal insertions of these gene fusions into *V. harveyi* were achieved by integrating the resultant suicide vectors via RecA dependent single homologous recombination as described previously (Fried et al., 2012). The conjugative plasmid transfer from donor strain *E. coli* WM3064 containing the required plasmid into *V. harveyi* was performed as described before. For this purpose, the donor and the recipient strain were cultivated in LB medium up to an OD_600_ of 0.8–1.0, supplemented with 300 μM *meso*-diaminopimelic acid (DAP) for the growth of *E. coli* WM3064. Single colonies were checked for chromosomal integration via performance of a PCR with the genomic DNA.

### Analysis of transcription levels via qRT-PCR

*Vibrio harveyi* ATCC BAA-1116 was cultivated as described above, and samples were harvested by centrifugation every hour. RNA was isolated as described before (Fritz et al., 2009). The RNA was used as template for random-primed first-strand cDNA synthesis according to the manufacturer's instructions. Quantitative real-time PCR (qRT-PCR) (iQ5 real-time PCR detection system, Biorad) was performed using the synthesized cDNA, a SYBR-green detection system (Biorad) and specific internal primers for *recA, luxN, luxQ, cqsS, luxO*, and *luxU* (Table S1). The CT value (cycle threshold) was determined after 40 cycles using the iQ software (Biorad). Values were normalized with reference to *recA* and relative changes in transcript levels were calculated using the comparative CT method (Schmittgen and Livak, 2008).

### Preparation of inverted membrane vesicles

*Escherichia coli* Rosetta (DE3) pLysS was transformed with plasmid pNKN, pNKQ, or pKK223-3-cqsS-F175C encoding wild type LuxN, LuxQ, or CqsS-F175C respectively. For overproduction the cells were induced using 1 mM IPTG. Membrane vesicles were prepared as described before and extensively washed to remove peripheral ATP (Timmen et al., 2006).

### Heterologous production of LuxP and LuxU

*Escherichia coli* MDAI-2 was transformed with the plasmid pGEX_LuxP, and LuxP was purified as described elsewhere (Neiditch et al., 2005). LuxU was overproduced using *E. coli* JM109 transformed with pQE30LuxU-6His, and purified as described in Timmen et al. (2006). All proteins were stored at −80°C.

### Phosphorylation assay

Phosphorylation reactions were performed in phosphorylation buffer (50 mM Tris/HCl pH 8.0, 10% (v/v) glycerol, 500 mM KCl, 2 mM DTT) at room temperature. The hybrid histidine kinases LuxQ, CqsS-F175C, and LuxN were used as full-length membrane integrated proteins in membrane vesicles. LuxQ containing membrane vesicles were added at final concentrations of 1 mg/ml. Via comparative Western Blot the amounts of LuxN and CqsS-F175C membrane vesicles needed were calculated as ratio based on LuxQ. The reaction mixture contained 0.36 mg/ml LuxU and 0.1 mg/ml LuxP unless otherwise indicated. To incorporate LuxP into LuxQ containing membrane vesicles, three cycles of freezing and thawing were performed. Unless otherwise indicated, AI-2 (synthesized by Rita Ventura, Instituto de Tecnologia Química e Biológica, Universidade Nova de Lisboa), HAI-1 (purchased from the University of Nottingham) and *V. cholerae* CAI-1 [*(S)* 3-hydroxytridecan-4-one*)* (kindly provided by Dr. Joachim Schultz, University of Tübingen) were added in a final concentration of 10 μM. The phosphorylation reaction was started by adding radiolabeled Mg^2+^-ATP, typically 100 μM [γ-^32^P] ATP (0.94 Ci/mmol; Perkin-Elmer, Rodgau-Jügesheim, Germany) and 110 μM MgCl_2_, and stopped at various time points by the addition of SDS loading buffer (Jung et al., 1997), followed by separation of the proteins on a SDS-PAGE (Laemmli, 1970). Gels were dried at 80°C on filter paper, exposed to a phosphoscreen for at least 24 h and scanned using a Typhoon Trio variable mode imager (GE Healthcare, München, Germany). Quantification of the bands was performed using ImageQuant (GE Healthcare, München, Germany).

For dephosphorylation assays, LuxU was phosphorylated using Lux(P)Q. In this case the phosphorylation buffer contained 10 mM CaCl_2_ instead of MgCl_2_ and twice the amount of Lux(P)Q and LuxU. After 10 min incubation at room temperature, membrane vesicles were removed by centrifugation (100,000 × *g*, 15 min, 4°C), and ATP and CaCl_2_ were removed by gel filtration (Sephadex G25 columns, GE Healthcare). Dephosphorylation of P-LuxU was started by the addition of 110 μM MgCl_2_, 100 μM ATP-γ-S (adenosine-5′-O-thiophosphate), and membrane vesicles containing the respective receptor. As described above, the reaction was stopped at the indicated time points, samples were subjected to SDS-PAGE and exposed to a phosphoscreen.

### In-gel fluorescence

Cells were cultivated as described above. In the early and late exponential growth phase (OD_600_ ~0.08 and ~0.7, respectively), cells were harvested by centrifugation (7,000 × *g*, 15 min, 4°C). The pellet was resuspended in TG buffer (50 mM Tris/HCl pH 8.0, 10% (v/v) glycerol) supplemented with lysozyme, DNase and PMSF (0.5 mM) and thereby concentrated 200 fold. After incubation at 37°C for 15 min, membranes were collected by ultracentrifugation (100,000 × *g*, 15 min, 4°C). The pellet was resuspended in TG buffer and diluted when indicated. Samples were mixed with buffer [200 mM Tris/HCl (pH 8.8), 20% (v/v) glycerol, 5 mM EDTA (pH 8.0), 0.02% (w/v) bromphenol blue (aliquots of 700 μl)], and before use 200 μl 20% (w/v) SDS and 100 μl 0.5 M DTT (Drew et al., 2006) was added. SDS-PAGE was performed according to Laemmli (Laemmli, 1970) and run at 150 V for 2 h in the dark to avoid bleaching of the fluorophore. In gel-fluorescence was analyzed using the Typhoon Trio scanner (Amersham Biosciences) with 488 nm laser and 526 nm emission filter. Quantification of fluorescence was performed using ImageQuant (GE Healthcare, München, Germany).

### Fluorescence microscopy

Cells were grown in AB medium until the late exponential growth phase and fluorescence microscopy was conducted. Three microliter of cells were spotted on 1% (w/v) agarose pad containing AB medium and imaged at 30°C. A DeltaVision Elite microscope (GE Healthcare, Applied Precision) equipped with a CoolSnap HQ2 CCD camera was used. Images were taken with 100x oil PSF U-Plan S-Apo 1.4 NA with an exposure time of 2 s using 475/28 nm excitation and 525/48 nm emission wavelengths. Analysis was performed using ImageJ.

## Results

### The QS receptors show differences in kinase but not phosphatase activities *in vitro*

*In vitro* phosphorylation assays were performed to determine the enzymatic activities of each QS receptor. For this purpose, genes of the full-length receptors were overexpressed; membrane vesicles were prepared and used for phosphorylation experiments. Previous characterizations of the kinase and phosphatase activities for LuxN and Lux(P)Q suggested that their kinase activities were in the same range and that the presence of AIs only influences the kinase but not the phosphatase activity (Timmen et al., 2006; Anetzberger et al., 2012). However, the AIs concentration dependent inhibition on the kinase activities was incomplete, indicating that even at high HAI-1 and AI-2 concentrations the phosphorylated LuxU was still detectable (Timmen et al., 2006; Anetzberger et al., 2012). Nonetheless, to quantitatively compare enzymatic activities we believe it is crucial to perform the assays for all QS receptors at the same time and under exactly same conditions. Since *V. harveyi* CAI-1 can only be enriched in dichloromethane, which interferes with our phosphorylation assay and CAI-1 is not commercially available, we introduced an amino acid substitution at the position 175 (Phe to Cys). *In vivo* studies have shown that the *V. harveyi* mutant CqsS-F175C exhibits relaxed specificity and detects both Ea-C8-CAI-1, the natural *V. harveyi* CAI-1, and the CAI-1 from *V. cholerae* (Ng et al., 2011). To ensure that equal protein amounts are used for the phosphorylation assays, we quantified the amount of the receptors in the membrane vesicles via Western blot.

**Figure 2:**
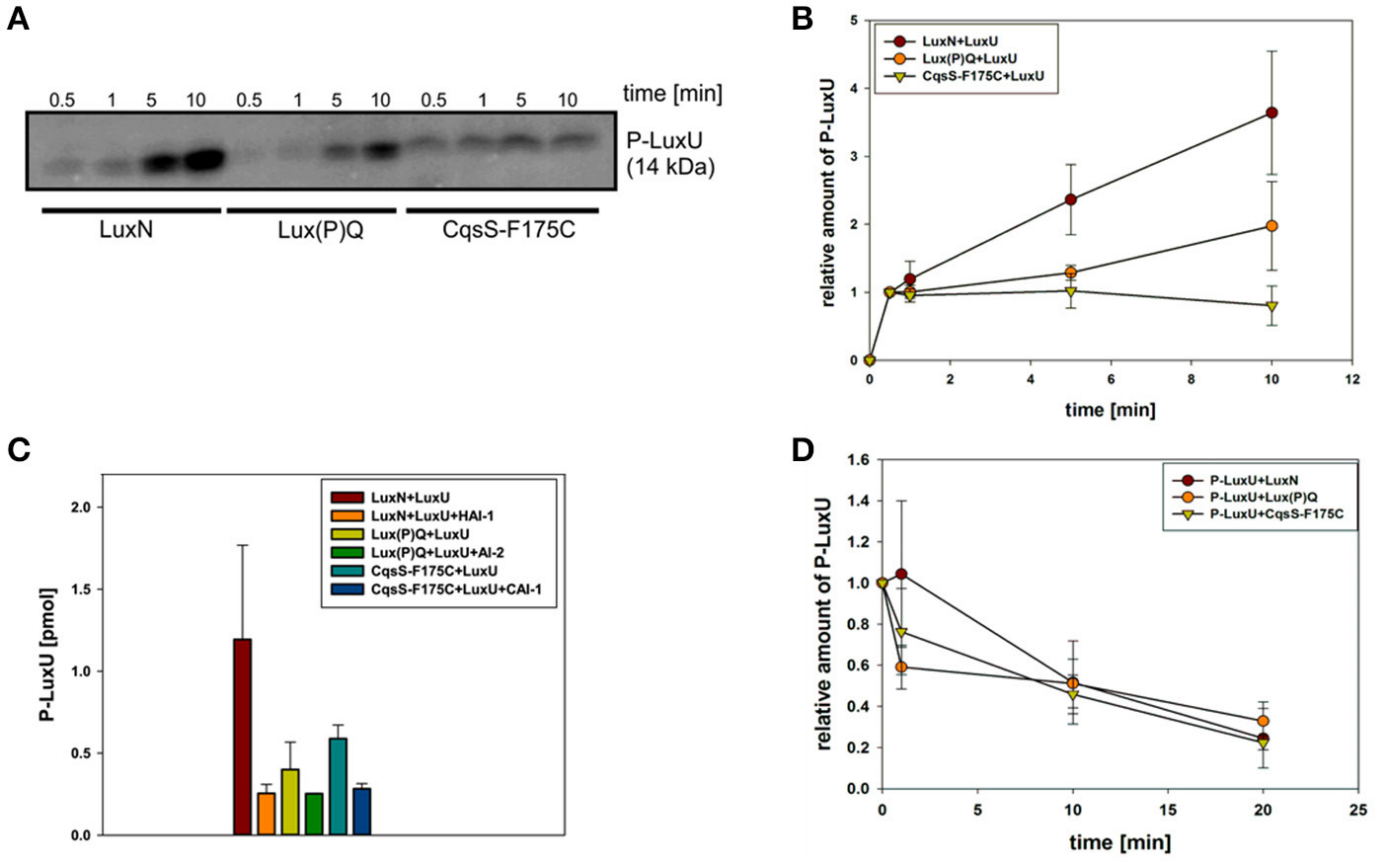
***In vitro*** **enzymatic activities of the QS receptors LuxN, Lux(P)Q, and CqsS-F175C. (A)** Time-dependent autophosphorylation of LuxN, Lux(P)Q and CqsS-F175C, and subsequent phosphotransfer to the HPt protein LuxU. Membrane vesicles containing one of the three QS receptors (all hybrid histidine kinases) LuxN, LuxQ, or CqsS-F175C, respectively, were mixed with LuxU, and the reaction was started with [γ^32^-P] Mg^2+^ATP. For LuxQ mediated phosphorylation, LuxP had been incorporated into the vesicles. At the indicated time points the phosphorylation reaction was stopped, proteins were separated by SDS-PAGE followed by exposure of the gels to a phosphoscreen. The autoradiograph corresponding to P-LuxU protein size is representative for three independent experiments. **(B)** Quantitative analysis of the data presented in **(A)**. Bands corresponding to P-LuxU were quantified using ImageQuant, and relative values were calculated (The amount P-LuxU at 0.5 min of phosphorylation was normalized to 1). **(C)** Influence of AIs on the kinase activity of the QS receptors. When indicated HAI-1, AI-2, or CAI-1 (from *V. cholerae*) was added to the assay mixture (described in **A**) in a concentration of 10 μM prior incubation. Ten minutes after incubation the reaction was stopped and P-LuxU was quantified. **(D)** Dephosphorylation of P-LuxU. A mixture of P-LuxU and LuxN, Lux(P)Q, or CqsS-F175C, respectively, was incubated. At the indicated time points the reactions were stopped, proteins were separated via SDS-PAGE, and the amount of P-LuxU was quantified. Relative values are presented, and the amount of P-LuxU in the absence of the hybrid histidine kinases was set to 1. All quantifications were performed with ImageQuant (GE Healthcare, Munich, Germany) using [γ^32^-P] Mg^2+^ATP as standard. Shown are the mean values and standard deviations of three independent experiments.

We have previously shown that autophosphorylation activity of LuxN is low and unstable, but the phosphorylation of the subsequent LuxU is stable (LuxU seems to act as a phosphate sink; Timmen et al., 2006). Therefore, we used the degree of phosphorylation of LuxU as a measure for the kinase activities of the QS receptors. LuxU cannot be phosphorylated by ATP (Timmen et al., 2006). The phosphorylation tube contained the corresponding QS-receptor and LuxU, and at time zero radiolabeled ATP was added to start the reaction. All three QS receptors autophosphorylated and phosphorylated LuxU in a time dependent manner (Figures 2A,B). The degree of increase of P-LuxU depends on the receptor present in the reaction (Figure 2B). After 10 min, the highest amount of P-LuxU was obtained in the presence of LuxN, indicating that LuxN presents the strongest kinase activity. Membrane vesicles containing CqsS-F175C showed a comparable initial rate of LuxU phosphorylation, however the saturation level of P-LuxU was already reached after 1 min (Figure 2B). Altogether the capacity of the receptors to phosphorylate LuxU showed the following order LuxN>Lux(P)Q>CqsS-F175C. Furthermore, the addition of each AI inhibited the respective kinase activity. It is interesting to note that despite the fact that LuxN shows the strongest kinase activity, all kinase activities were inhibited to the same degree upon AI addition (Figure 2C). However, the inhibitory effects of all three AIs were incomplete and residual kinase was still detected, which is in agreement with previous studies (Anetzberger et al., 2012).

In order to investigate the corresponding phosphatase activities, LuxU was phosphorylated with [γ^32^-P] ATP via Lux(P)Q, purified and employed for subsequent dephosphorylation analysis. All three receptors were able to dephosphorylate P-LuxU in a time-dependent manner (Figure 2D). P-LuxU is stable for at least 40 min (Timmen et al., 2006), excluding the possibility that the decrease of P-LuxU is due to the instability of the protein and/or the phosphorylation (Figure S1). The dephosphorylation of P-LuxU was calculated as number of fold-reduction based on P-LuxU incubated without the receptors. All three receptors dephosphorylated the P-LuxU to a comparable level in a similar timeframe (Figure 2D). However, none of the hybrid histidine kinases was able to dephosphorylate P-LuxU completely.

### Receptor transcript levels vary during growth phases

**Figure 3:**
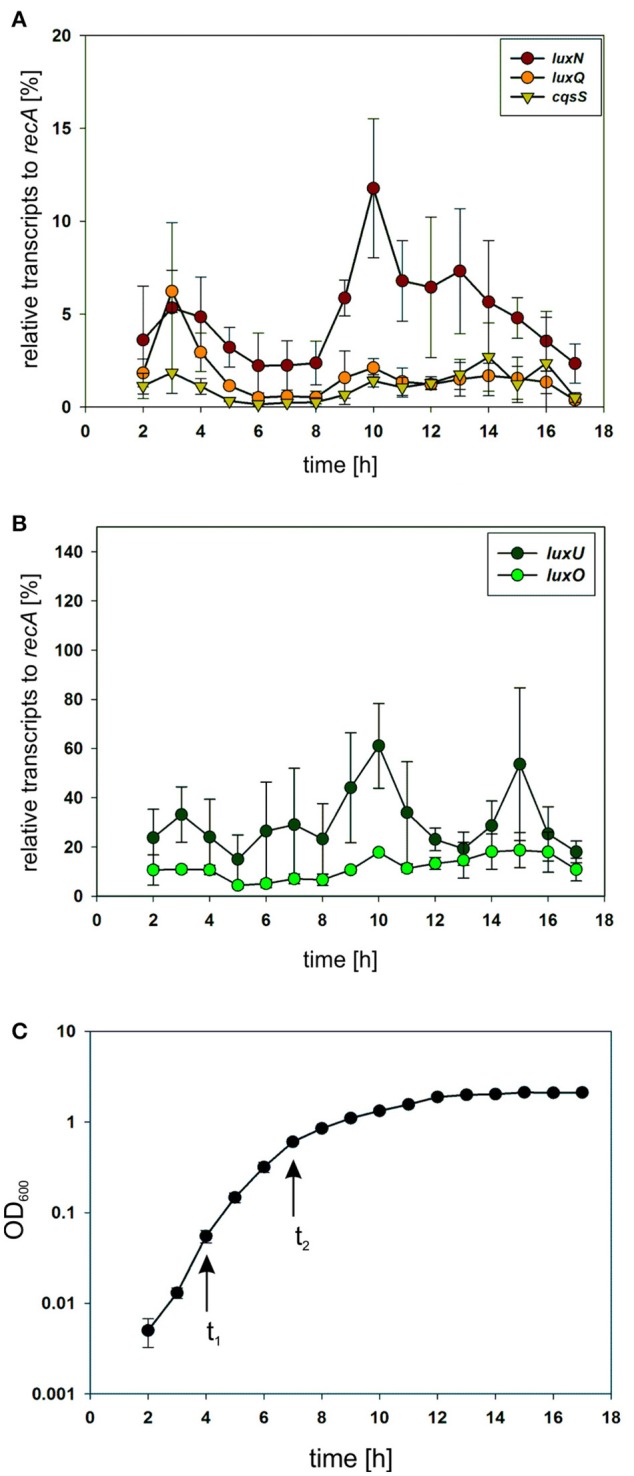
**(A,B)** Transcriptional analysis of the components of the QS cascade in *V. harveyi*. Cells of an overnight culture of *V. harveyi* were diluted 5,000 times in fresh AB medium and cultivated aerobically at 30°C. Samples were taken every hour and total RNA was isolated. Transcript levels of *luxN, luxQ, cqsS, luxU*, and *luxO* were determined via qRT-PCR. Transcript levels relative to *recA* were calculated using the C_*t*_ method (Schmittgen and Livak, 2008). Experiments were performed in triplicates and error bars represent the standard deviation of the mean. **(C)** Optical densities (OD_600_) determined for *V. harveyi* corresponding to time points for RNA extraction. t_1_ and t_2_ indicate time points used for further experiments.

To investigate the potential differences in the quantity of the QS components, we performed qRT-PCR and compared transcript levels of the QS receptors. Transcript levels were measured from the *V. harveyi* wild type by taking samples of the cell culture every hour (Figure 3C). Overall, *luxN* was the most transcribed kinase with its highest transcript levels in the late exponential growth phase (Figure 3A). The transcription of *luxQ* and *cqsS* did not differ significantly and, except for a slight peak for *luxQ* after 3 h, remained constant (Figure 3A). Moreover, we could show that the transcript levels of the kinases were overall lower compared to *luxU* coding for the HPt protein, or *luxO* coding for the response regulator at any time (Figures 3A,B). The transcript levels of *luxU* were 2-fold higher than the mRNA levels of *luxO* (Figure 3B).

### The QS receptors are clustered in *V. harveyi* and differ in their abundance

In many bacteria, chemotaxis receptors form clusters at the cell poles to detect chemical signals from the environment with high sensitivity over a wide range of concentrations (Briegel et al., 2009). In order to elucidate the potential cluster formation of the QS receptors we performed electron cryotomography (ECT) with the wild type *V. harveyi* and a triple kinase mutant (*V. harveyi* Δ*luxN*Δ*luxQ*Δ*cqsS)*. This ECT method was successfully used to identify cluster formation for many bacterial chemoreceptors (Briegel et al., 2009). However, we could not find any differences between the wild type and the triple mutant (Ariane Briegel and Grant Jensen, data not shown). This could be either due to the low receptor expression levels or to the lack of cluster formation.

Subsequently, we constructed reporter strains encoding full-length QS receptors tagged with a C-terminal fluorophore to investigate their subcellular localization as well as the corresponding protein levels. All genes encoding these fluorophore hybrids were integrated at the native locus in the chromosome. Our first attempts to visualize the receptors using GFP or mCherry fusion protein were unsuccessful presumably due to the low expression levels. Contrarily, the receptors fused to the mNeonGreen (NG) protein showed fluorescence signal at the membrane, most likely due to the higher brightness compared to e.g., GFP (Shaner et al., 2013). Cells were grown until late exponential growth phase (see Figure 3C t_2_) and subsequently employed for fluorescence imaging on agar pads.

**Figure 4:**
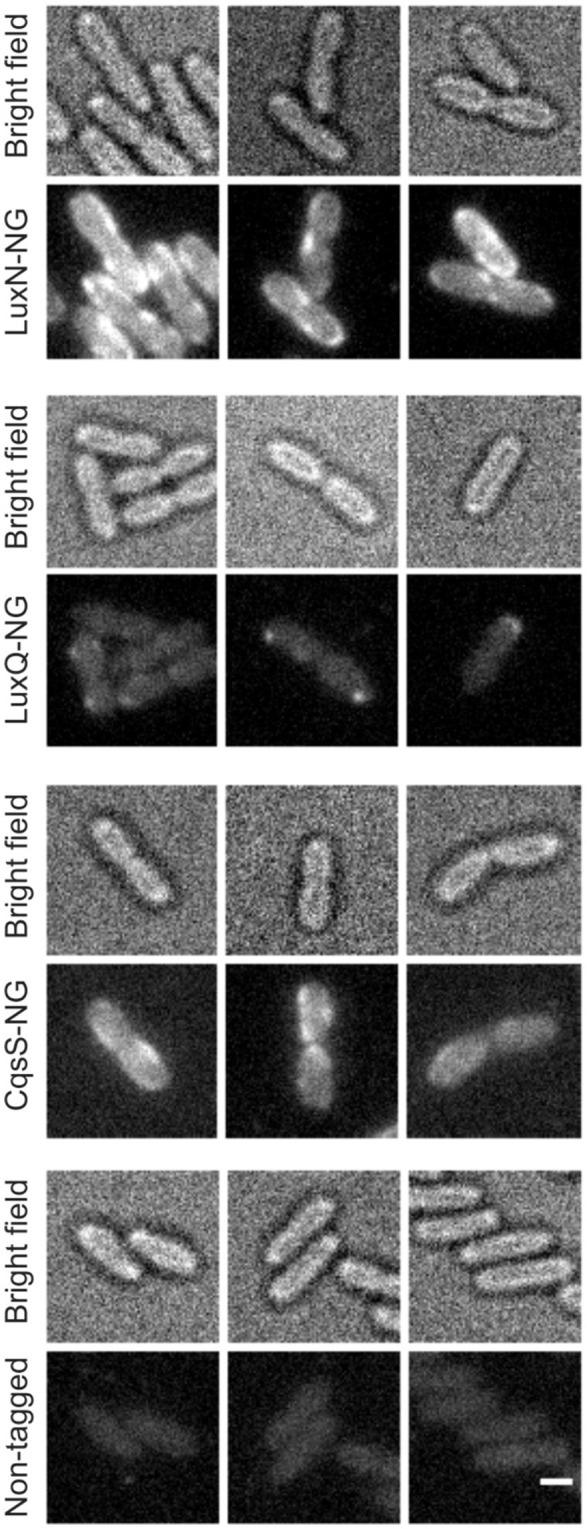
**Localization of the QS receptors**. Bright field (always upper row) and fluorescence microscopy (lower row) images of cells producing LuxN-NG, LuxQ-NG, CqsS-NG, or the non-tagged wild type (from top to bottom). Fluorescence microscopy (exposure time 2 s) was performed using cells in the late exponential growth phase and immobilized on 1% (w/v) agarose pads containing AB medium. Protein-fluorophore hybrids are indicated by increased fluorescence compared to background. Scale bar 1 μm.

The non-tagged *V. harveyi* wild type was used as control for background fluorescence to ensure that the observed signals are not artifacts (Figure S2). The signal in the control was significantly lower compared to the reporter strains harboring the receptor fluorophore hybrids. LuxN-NG clustered in the membrane and exhibited the strongest fluorescence signal (Figure 4 upper panel). LuxQ-NG also formed clusters, however, LuxQ-NG localized preferentially at the cell poles (Figure 4 second panel from top). Contrary to LuxN and LuxQ proteins, the fluorescence of CqsS-NG was rather homogeneously distributed in the membrane (Figure 4 third panel from top). The negative control with the non-tagged wild type using the same imaging settings confirmed that the clusters seen in the reporter strains were not artifacts (Figure 4 bottom panel).

**Figure 5:**
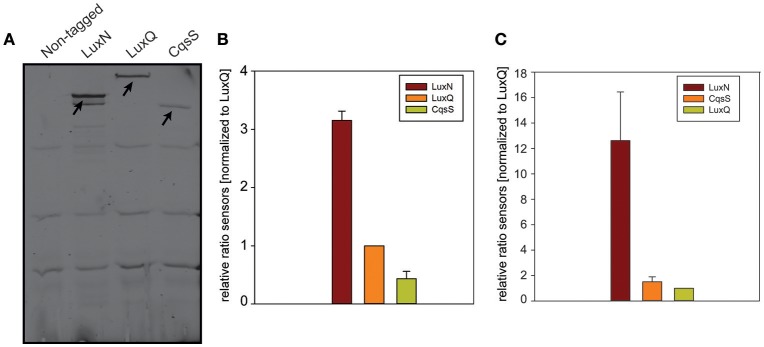
**In-gel fluorescence. (A)** SDS-PAGE of membrane fractions of the non-tagged wild type or cells harboring LuxN-NG, LuxQ-NG, or CqsS-NG harvested in the early exponential growth phase (time point t_1_ Figure 3C). Cell densities were adjusted (OD_600_ 0.08) and ~75 μg protein were loaded for each strain. SDS-PAGE was run for 2 h at 150 V in the dark, and the gel was scanned using the Typhoon Trio scanner (Amersham Biosciences) with 488 nm laser and 526 nm emission filters. Arrows indicate the bands corresponding to LuxN-NG, LuxQ-NG, and CqsS-NG. Gel images are representative of three independent experiments. **(B)** Abundance of LuxN-NG, LuxQ-NG, CqsS-NG in the early exponential growth phase (t_1_ Figure 3C). Band intensities were quantified using ImageQuant. All values were normalized to LuxQ and therefore represent receptor-fluorophore abundance as a factor of LuxQ abundance. **(C)** Abundance of LuxN-NG, LuxQ-NG, CqsS-NG in the late exponential growth phase (t_2_ Figure 3C). Band intensities were quantified using ImageQuant. All values were normalized to LuxQ and therefore represent receptor-fluorophore abundance as a factor of LuxQ abundance. Bars in **(B,C)** represent mean values from three independent experiments and standard deviations of the mean are indicated.

To determine the abundance of each of the receptors, we performed in-gel fluorescence assays. For this assay, we used the same reporter strains as for the microscopy approach. Cells were harvested in the early and late exponential growth phase, respectively (for time points see Figure 3C t_1_ and t_2_), lysed, the membrane fractions were prepared and subsequently analyzed by SDS-PAGE. For each reporter strain (LuxN-NG, LuxQ-NG and CqsS-NG) a specific band (corresponding to the size of the hybrid receptor) could be observed, which was absent in the non-tagged wild type control (Figure 5A). In the early exponential growth phase LuxN was the most abundant kinase, followed by LuxQ and CqsS (Figure 5A). Quantification of the band intensities using ImageQuant and normalized to the LuxQ band showed that the amount of LuxN was three times higher than LuxQ. Whereas CqsS showed the lowest copy number and was two-fold lower compared to LuxQ (Figure 5B). In the late exponential growth phase, specific bands for each of the fusions could be observed as well. However, the distinct expression pattern differed from the one in the early exponential phase. LuxN was still the predominantly expressed kinase, but its ratio relative to the other kinases was further increased: LuxN was more abundant than LuxQ by a factor of 8 ± 3. LuxQ and CqsS showed comparable protein amounts with CqsS being slightly more abundant than LuxQ (factor 1.24 vs. 1; Figure 5C). It is interesting to note, that the same differences on the abundance of the receptors were observed also at the transcript levels (compare Figures 3, 5).

## Discussion

Sensing and integration of multiple environmental signals are important for adaption and different behaviors of bacteria. For example, *E. coli* integrates positive and negative signals to generate an integrated chemotactic response (Khan et al., 1995). In the case of cell-to-cell communication, several bacterial species are known to synthesize and respond to multiple AIs/environmental signals. *V. cholerae* integrates four inputs in one cascade (Jung et al., 2015). *Vibrio harveyi* perceives three different AIs (AI-2, HAI-1, and CAI-1) with three different receptors, specifically hybrid histidine kinases (Lux(P)Q, LuxN, and CqsS), and integrates the information in one signaling cascade, which affects the expression of more than 100 genes (Waters and Bassler, 2006). AI-2 is produced by various bacterial species, while HAI-1 is solely synthesized and sensed by *V. harveyi*. CAI-1 with an 8-carbon hydrocarbon tail is perceived by *V. harveyi* CqsS, whereas *V. cholerae* CqsS can perceive CAI-1 with an 8- or 10-carbon hydrocarbon tail. Accordingly, these systems are supposed to be specific for inter-species, intra-species, and intra-genus communication (Federle and Bassler, 2003; Henke and Bassler, 2004; Ng et al., 2011). To monitor the availability of the AIs it is crucial that the hybrid histidine kinases exhibit two enzymatic activities: kinase and phosphatase. It is suggested that this bifunctionality ensures robustness in the output response (Siryaporn et al., 2010). Moreover, we have shown that the ratio of the kinase and the phosphatase activities of the different receptors, and hence the extent of phosphorylated LuxU and LuxO is crucial for the output strength and for the degree of phenotypic heterogeneity (Plener et al., 2015).

Here, we comparably characterized the enzymatic activities of each individual QS receptor. While the initial rates of autophosphorylation and phosphotransfer to LuxU were similar among the receptors, LuxN showed the highest capacity to phosphorylate LuxU followed by Lux(P)Q and CqsS-F175C (Figures 2A,B). Due to methodological reasons (non-availability of Ea-C8-CAI-1), we characterized the enzymatic activities of the CqsS variant CqsS-F175C, which responds to the CAI-1 from *V. cholerae* (Ng et al., 2011). *In vivo* and *in vitro* studies have shown previously that certain single amino acid replacements in CqsS of *V. harveyi* or *V. cholerae* alter their ligand specificities, without significantly affecting their signaling activities (Ng et al., 2011; Wei et al., 2012). Therefore, it was not surprising that the kinase activities of all three QS-receptors were inhibited in the presence of their corresponding AIs. However, the comparison revealed that the presence of AIs reduced the amount of P-LuxU generated by each AI/receptor pair to the same level (Figure 2C) even in case of LuxN, which was able to phosphorylate LuxU to a higher degree in the absence of HAI-1. It is interesting to note, that none of the three AI/receptor pairs was able to completely inhibit the phosphorylation of LuxU (Figure 2C), which is also consistent with earlier studies (Timmen et al., 2006; Anetzberger et al., 2012; Wei et al., 2012). The latter result indicates that each of the three receptors retains residual kinase activity despite of the ligand binding. Although this phenomenon is mechanistically difficult to explain at the protein level, this result is in agreement with *in vivo* observations: (i) even at HCD (at high CAI-1 concentration), low levels of *qrr*1-4 transcripts are detectable in *V. cholerae* (Rutherford et al., 2011), and (ii) only in the presence of all AIs the induction of bioluminescence was homogeneous in the *V. harveyi* population (Plener et al., 2015). All three QS receptors also exhibited phosphatase activity, which is crucial upon accumulation of the AIs to switch the QS cascade to an ON state by draining the phosphoryl groups out of the cascade. In contrast to the differences observed in kinase activities, we neither found differences in phosphatase activities among the three receptors (Figure 2D) nor was this activity influenced by the AIs (data not shown). In agreement with similar studies (Timmen et al., 2006; Anetzberger et al., 2012; Wei et al., 2012), our results show that the phosphatase activity of LuxN, Lux(P)Q, and CqsS-F175C is not regulated by the AIs and ensures constant dephosphorylation of P-LuxU. In summary, the enzymatic activities of the three QS receptors tested here are very similar, except that their capacity to phosphorylate LuxU differs in the order of LuxN>Lux(P)Q>CqsS-F175C. We speculate that the higher phosphorylation capacity of LuxN is advantageous for *V. harveyi* to respond to a wide dynamic range of the species-specific AI.

In addition to the inherent enzymatic activities, the abundance of each QS receptor might affect the QS output. For this purpose, we first investigated the transcript levels of all components of the QS cascade and could show that the genes of the QS receptors are transcribed to a lower extent in comparison to *luxU* (encoding the HPt protein) or *luxO* (encoding the response regulator) (Figure 3). These results highlight the function of LuxU and LuxO to collect all information from the three QS receptors. In comparison to the other QS receptors, we found the highest mRNA level for *luxN* with a peak after 10 h of cultivation (Figure 3A). This result is consistent with the increase of *luxN* transcription at higher HAI-concentrations, which alleviate the negative feedback loop mediated by the Qrr sRNAs (Teng et al., 2011; Figure 1). It was shown before, that the three AIs are not produced at the same time, and rather follow a distinct pattern: AI-2 is produced in the early exponential growth phase followed by HAI-1 and CAI-1 in the late exponential phase (Anetzberger et al., 2012). Hence, upon accumulation of AIs, meaning less expressed Qrr sRNAs, the *luxMN* transcripts are no longer degraded and the number of *luxN* transcripts increases. For the *luxQ* transcripts, we observed a small peak as well, however there has not been described any feedback loop that could explain this increase in mRNA levels (Figure 3A). The increase of LuxN copy numbers regulates the sensitivity of *V. harveyi* for HAI-1. It was demonstrated before, that cells with a constitutive high LuxN copy number became almost insensitive to AI-2 (Teng et al., 2011).

In addition, we constructed various reporter strains harboring mNeonGreen (NG) tagged QS receptors (LuxN-NG, LuxQ-NG, CqsS-NG). After imaging the cells in the late exponential growth phase, we could observe differences in the relative abundance among the three receptors. LuxN seemed to be the most abundant receptor compared to CqsS and LuxQ (Figure 4). Furthermore, we observed differences in their subcellular organization. We found LuxN in larger clusters compared to LuxQ, but these clusters were not solely located at the cell poles. LuxQ in turn formed small spots preferentially localized close to the cell poles. CqsS seemed to be not specifically located, but distributed along the membrane (Figure 4). Using these fluorescently labeled QS receptors, we could also determine their abundance. Interestingly, already in the early growth phase LuxN was more abundant (factor 3 compared to LuxQ) than LuxQ or CqsS (factor ~0.5 compared to LuxQ) (Figure 5). As expected and due to alleviation of the negative feedback loop (Teng et al., 2011), the LuxN copy number further increased (LuxN factor ~8 compared to LuxQ), while CqsS protein levels were slightly elevated compared to LuxQ in the late exponential growth phase (Figure 5). The negative feedback loop on *luxMN* relies on the presence of the sRNAs. Consequently, increasing concentrations of AIs at higher cell densities lead to less sRNAs and therefore elevated amounts of *luxN* transcripts and finally higher levels of LuxN.

It is worth mentioning here, that a *V. harveyi* mutant in which only LuxN is present (Δ*cqsS*, Δ*luxQ*), produces a low level of bioluminescence arguing for either a weak phosphorylation of LuxU/LuxO or a low LuxN copy number (Plener et al., 2015). Our new results clearly do not support this assumption and raise the question, whether the enzymatic activities of the QS receptor LuxN are influenced by unknown factor(s) in whole cells.

In bacterial chemotaxis, clustering of chemoreceptors ensures amplification of the signal and integration of multiple signals with a wide dynamic range (Maddock and Shapiro, 1993; Sourjik, 2004). Thereby the cells respond to even small changes in the concentration of an attractant or repellant (Sourjik, 2004). Moreover, the extracellular chemical stimuli are sensed either by direct binding of the ligands to the receptor or via indirect binding of a periplasmic binding protein with bound ligand (Neumann et al., 2010). Direct binding of the ligands leads to a higher dynamic range, while binding via periplasmic binding proteins results in high sensitivity and a narrower dynamic range (Neumann et al., 2010).

We did not find an arrangement of the QS receptors in an array, suggesting that they work in parallel and transduce the AIs information without amplification of the signals and without mutual interference into the cell. Furthermore, HAI-1 and CAI-1 directly bind to LuxN and CqsS, respectively, while the perception of AI-2 is dependent on the periplasmic binding protein LuxP. This argues that the bacteria-specific AI-2 signal is sensed with high sensitivity, but only in a narrow concentration range. In contrast, *V. harveyi* has developed the widest dynamic range of sensing for HAI-1, the species-specific signal. Here, we could show that LuxN has not only the highest phosphorylation capacity for LuxU, but LuxN also showed higher abundance, and its copy number adapts during the cell growth. The feedback loop on LuxN numbers might help *V. harveyi* to focus on HAI-1 signal and/or to monitor its developmental stage (Mehta et al., 2009). CqsS seems to play a minor role (low copy number), but certainly also contributes to QS by recognizing CAI-1 and thereby inhibiting its kinase activity.

Altogether, our data contribute to a better understanding of receptor-mediated QS signaling cascades by bringing both qualitative (relative activity of the receptors) and quantitative (abundance of the receptors) approaches together.

## Author contributions

NL, JS, and KJ planned the experiments and analyzed all data. NL performed qRT-PCR, overproduced all proteins and conducted all *in vitro* assays. NL generated all reporter strains, and performed microscopy as well as in gel fluorescence. JS established the in-gel fluorescence and the fluorescence microscopy, and quantitatively analyzed data derived from fluorescence microscopy. NL and KJ wrote the manuscript.

### Conflict of interest statement

The authors declare that the research was conducted in the absence of any commercial or financial relationships that could be construed as a potential conflict of interest.
