# Supplementary material for: Activity, Abundance, and Localization of Quorum Sensing Receptors in Vibrio harveyi
Source: Front Microbiol. 2017 Apr 18;8:634. doi: 10.3389/fmicb.2017.00634 (PMC5394107; doi:10.3389/fmicb.2017.00634)
Supplement: Supplementary file 1 [file DataSheet1.DOCX]

**Supplemental Material**

Activity, abundance and localization of quorum sensing receptors in *Vibrio harveyi*

Nicola Lorenz^1^, Jae Yen Shin^1^ and Kirsten Jung^1*^.

^1^Munich Center for Integrated Protein Science (CIPSM) at the Department of Biology I, Microbiology, Ludwig-Maximilians-Universität München, Martinsried, Germany

**
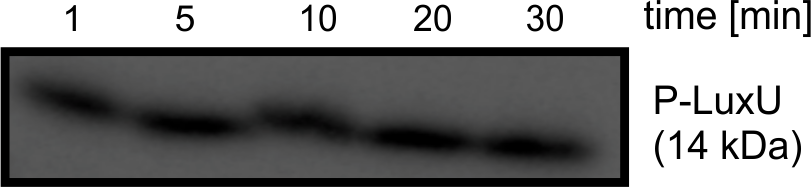
**

**Figure S 1 Stability of P-LuxU *in vitro.*** LuxU was initially phosphorylated via Lux(P)Q with [γ^32^-P] Mg^2+^ ATP. After purification, P-LuxU was incubated at room temperatures for distinct times. At the indicated times the phosphorylation reaction was stopped, proteins were separated by SDS-polyacrylamide gel electrophoresis followed by exposure of the gels to a phosphoscreen. The autoradiograph corresponding to LuxU protein size is representative of three independent experiments.

**
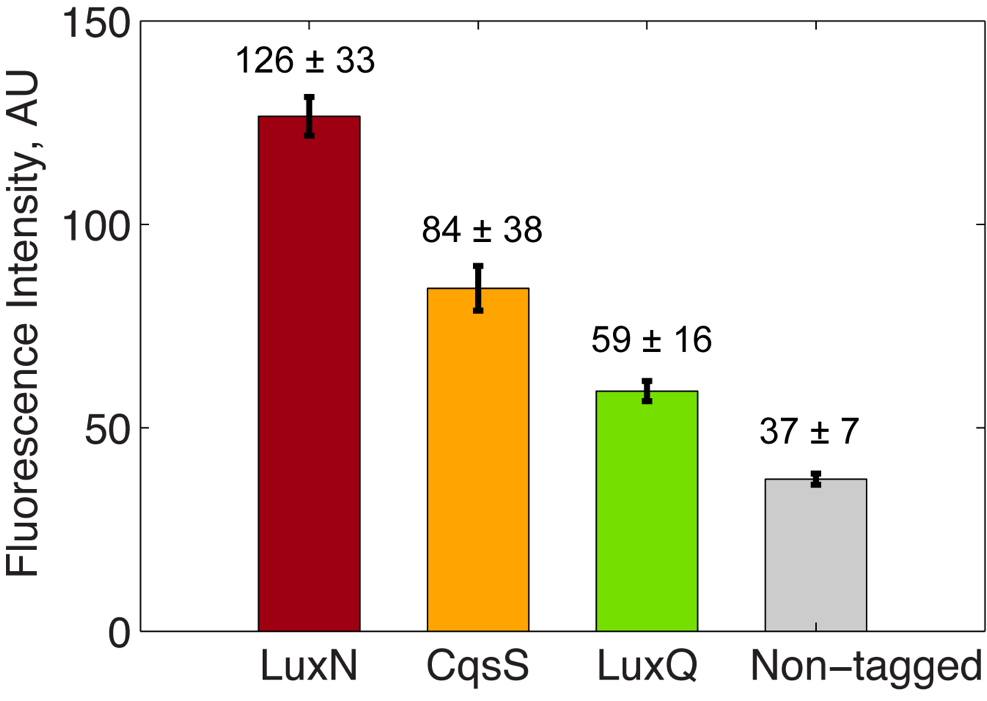
**

**Figure S 2. Green fluorescence intensity of strains expressing the hybrid proteins LuxN-, CqsS-, or LuxQ-mNeonGreen, and the background strain (Non-tagged) determined by using the software Fiji.** These results indicate that the relative abundance of the receptors decreases in a following manner: LuxN > CqsS > LuxQ. All three receptors (LuxN, CqsS and LuxQ) present significantly higher fluorescence intensity compared to the non-tagged strain (P<0.0001, extremely statistically significant). Values of the mean and the standard deviation of the fluorescence intensity are shown in the graph. The numbers of analyzed cells were respectively 50, 48, 41 and 26, for the LuxN-, CqsS- and LuxQ- mNeonGreen expressing strains, and the background strain.

**Table S 1 Primers used in this study**

| **Name** | **Sequence** |
| --- | --- |
| Fluorophor fusions |  |
| mNeonGreen PspOMI s | CCGGGCCCATGGTGAGCAAGGGCGAGGAGGAT |
| mNeonGreen SpeI as | CCACTAGTTTACTTGTACAGCTCGTCCATGCC |
| LuxN sense 500 bp up | CGCGATACTTGGCTCAGCTACGCGCCA |
| LuxN BamHI ohne Stop as | CCGGATCCTTCTCTCTCAGCTTCACAAGC |
| LuxQ +300 bp up BamHI s | CCGGATCCCGACGTAGCATTAGGTGCA |
| LuxQ ohne stop PspOMI as | CCGGGCCCGGTTCTTTCTACCAAGAA |
| Up CqsS +500 bp BamHI s | CCGGATCCGCTCGATTAGATGCATGGTTT |
| cqsS PspOMI as | TAGGGCCCAATCCAGTTCGCAATCTTGTC |
| CqsS overproduction |  |
| cqsS EcoRI s | GATAAAGAATTCATGGACGCGATTCGCAAAGTATATCAG |
| CA cqsS HindIII as | CAAGAGAAGCTTCTAATGATGATGATGATGATGAATCCAGTTCGCAATCTTGTCG |
| CqsSF175C_s | TTGGTAACTTGTGCTACTTCCGA |
| CqsSF175C_as | TTTCGGAAGTAGCACAAGTTACC |
| qRT PCR |  |
| luxN 142 s | GCAGCTTATATTGCGTACTCGGTGTG |
| luxN 293 as | GCAAAAGCAAATGCGAAGAAGGAAGC |
| luxQ 197 s | GCCACTTTGCGGCGATCCAGATACA |
| luxQ 353 as | GTTAGAAAACGGAATTCTGGTGTGTGC |
| cqsS 217 s | GCCTATTATCAAGTGGTCACAACGCT |
| cqsS 363 as | CATCACCGATGTGATATGCACGAGC |
| recA 240 s | GCTAACTCTTGAGCTTATTGCTGCTG |
| recA 395 as | AGCGCTTGCTCACCTGTGTCTGGC |
| Sequencing |  |
| M13_uni -21 | GTAAAACGACGGCCAGT |
| M13_reverse -29 | AACAGCTATGACCATG |
| CqsS_V456_s | TTCTCGATGTTCCTGCGGTTC |
| CqsS_L223_s | TTAAGTGATGAAGATGTGAC |
| pKK176 | AAATCACTGCATAATT |
